# Radiosurgery for pituitary adenomas: evaluation of its efficacy and safety

**DOI:** 10.1186/1748-717X-5-109

**Published:** 2010-11-17

**Authors:** Douglas G Castro, Soraya AJ Cecílio, Miguel M Canteras

**Affiliations:** 1 Institute of Neurological Radiosurgery (IRCN), Alvorada street, 64, suit 13/14, São Paulo-SP, ZIP: 04550-000, Brazil

## Abstract

**Object:**

To assess the effects of radiosurgery (RS) on the radiological and hormonal control and its toxicity in the treatment of pituitary adenomas.

**Methods:**

Retrospective analysis of 42 patients out of the first 48 consecutive patients with pituitary adenomas treated with RS between 1999 and 2008 with a 6 months minimum follow-up. RS was delivered with Gamma Knife as a primary or adjuvant treatment. There were 14 patients with non-secretory adenomas and, among functioning adenomas, 9 were prolactinomas, 9 were adrenocorticotropic hormone-secreting and 10 were growth hormone-secreting tumors. Hormonal control was defined as hormonal response (decline of more than 50% from the pre-RS levels) and hormonal normalization. Radiological control was defined as stasis or shrinkage of the tumor. Hypopituitarism and visual deficit were the morbidity outcomes. Hypopituitarism was defined as the initiation of any hormone replacement therapy and visual deficit as loss of visual acuity or visual field after RS.

**Results:**

The median follow-up was 42 months (6-109 months). The median dose was 12,5 Gy (9 - 15 Gy) and 20 Gy (12 - 28 Gy) for non-secretory and secretory adenomas, respectively. Tumor growth was controlled in 98% (41 in 42) of the cases and tumor shrinkage ocurred in 10% (4 in 42) of the cases. The 3-year actuarial rate of hormonal control and normalization were 62,4% and 37,6%, respectively, and the 5-year actuarial rate were 81,2% and 55,4%, respectively. The median latency period for hormonal control and normalization was, respectively, 15 and 18 months. On univariate analysis, there were no relationships between median dose or tumoral volume and hormonal control or normalization. There were no patients with visual deficit and 1 patient had hypopituitarism after RS.

**Conclusions:**

RS is an effective and safe therapeutic option in the management of selected patients with pituitary adenomas. The short latency of the radiation response, the highly acceptable radiological and hormonal control and absence of complications at this early follow-up are consistent with literature.

## Introduction

Pituitary adenomas represent nearly 15% of all intracranial tumors and are associated with significant morbidity due to either local compressive effects and/or hormonal hypersecretion [1]. Their clinical classification into non-functioning or functioning tumors is defined on the basis of hormonal serum level. Surgery, radiotherapy and medication are the three key elements of the treatment strategy [2]. Transsphenoidal microsurgery has remained the primary treatment for most patients with non-functioning pituitary microadenomas or functioning microadenomas causing acromegaly or Cushing's disease. Most prolactinomas can be controlled succesfully by medical treatment and transsphenoidal microsurgery is the second treatment step [3].

The persistence or recurrence of disease due to tumor invasion into surrounding structures or incomplete tumor resection is quite common and long term tumor control rates after transsphenoidal excision alone vary from 50 to 80%[4]. For residual or recurrent tumors fractionated radiation therapy has been the traditional treatment. However, it has a prolonged latency up to one decade for its effects and is associated with more frequent side effects as hypopituitarism, visual damage and cerebral vasculopathy [2,5].

Recently, radiosurgery (RS) has gained acceptance as a complementary treatment option in combination with microsurgery. RS provides growth control and long-term endocrine control that is superior to that of repeat resective surgery and the latency of the radiation response is substantially shorter than that of fractionated radiotherapy. Besides that, as RS better limits radiation exposure of the surrounding normal brain, it has been associated with a significantly lower morbidity than conventional fractionated radiotherapy [5-8].

This investigation was conducted to evaluate the effects of Gamma Knife RS on the growth and endocrinological response and its safety in the treatment of pituitary adenomas.

## Materials and methods

### Patient population

Forty-two out of the first 48 consecutive patients with pituitary adenoma were treated with RS at the Institute of Neurological Radiosurgery between 1999 and 2008, with a 6 months minimum follow-up.

Radiosurgery was delivered as a primary or adjuvant treatment. There were 14 patients with non-secretory adenomas (NSA) and, among functioning adenomas, 9 were prolactinomas (PRL), 9 were adrenocorticotropic (ACTH) and 10 were growth hormone-secreting tumors (GH).

All study patients met the eligibility criteria: histological or radiological diagnosis of pituitary adenoma and a minimum 3 mm distance between the tumor and optic apparatus. In patients selected for RS, clinical, laboratorial and radiological evaluation were performed. Clinical evaluation consisted of neurological examination and a comprehensive ophthalmological evaluation including visual field tests. Laboratorial and radiological evaluation included hormonal level assessment and magnetic resonance imaging (MRI).

### Treatment

RS was performed using the Leksell gamma unit model B (Elekta Instruments; Atlanta, GA, USA) with 201 ^60^Co sources.

Images for target definition and dose planning were obtained from both MRI and computerized tomography scanning (CT). The MRI studies were T1-weighted 1 mm axial and coronal gadolinium-enhanced slices and T2-weighted 1 mm coronal slices. The CT images consisted of a series of contrasted-enhanced 1 mm slices. The images were exported to GammaPlan V2.01 (Elekta Instruments; Atlanta, GA, USA) for dose planning. Microadenomas usually appear as hypointense lesions on T1-weighted MRI. The gadolinium contrast to adjacent normal gland enhances and highlights the injury. Macroadenomas are usually isointense on T1 and enhance homogeneously, but more slowly than normal tissue. Dose selection was limited by the tolerance of the adjacent structures. The maximum dose applied to the optic nerves and chiasm was most frequently 8 Gy and was rarely as high as 9 Gy. Functioning tumors received the highest possible marginal dose, as higher marginal dose are associated with a higher rate of hormonal normalization. A minimum marginal dose of 12 Gy was generally considered for non-functioning tumors.

### Therapeutic evaluation criteria and follow-up

We defined hormonal control (HC) as the junction of hormonal normalization (HN) and hormonal response (HR). The latter was defined as a decline in the measured hormonal level of more than 50% from the pre-RS hormonal levels. In order to define HN, in ACTH-secreting tumors, we used the dosage of ACTH serum as a parameter. In GH-secreting, we evaluated the basal GH and IGF-1 and appropriate sex and age, in prolactinomas, we consider the level of serum prolactin and appropriate sex. Radiological control (RC) was defined as the junction of radiological stasis (RSt) and radiological response (RR). RSt was defined as a tumor enlargement or shrinkage of less than 20% and RR as a tumor shrinkage of more than 20%.

Pituitary deficiency was defined as a requirement for new hormonal replacement medication after RS or a requirement for a dose increase in preexisting hormone therapy. Visual deficit due to RS was regarded if the patient reported post-RS visual complaints related to damage to the perisellar optic apparatus confirmed by visual field and acuity examinations.

The follow-up schedule included clinical examinations with ophthalmological and endocrinological evaluations and MRI of the brain and sellar region at 6-month intervals for the first 24 months after treatment and annually thereafter.

### Statistical analysis

All statistical analyses were performed with a statistical software package (SPSS version 13.0; SPSS Inc; Chicago, IL, USA). Cumulative rates for HC and HN were calculated using the Kaplan-Meier method. Univariate analysis was assessed using the log-rank-test. Differences were considered statistically significant at p < 0.05.

## Results

### Patient characteristics

The median follow-up period was 42 months (6-109 months). The median patient age at the time of the procedure was 43 years (range 16-78 years). There were 20 men (48%) and 22 women (52%). Most patients were treated for residual (76%) or recurrent tumors (17%) after surgery, medication or radiotherapy, whereas only 3 patients (2 patients with prolactinomas and 1 patient with non-functioning adenoma) had RS as a primary treatment (7%). Before RS, surgery alone was done in 22 patients, medication alone in 5 and both treatments in 12 patients. Only 2 patients were treated with surgery followed by external beam radiotherapy (EBRT). Before RS, 17 patients used medication while 13 patients used it after RS.

### Treatment characteristics

The median target volume was 1.3 cm^3 ^(range 0.03-11.1 cm^3^). Multiple isocenters ranging from 1 to 16 in number (median 7) were used, resulting in the median conformity index of 0.89 (range 0.42-1.7). The tumor margin was covered by an isodose ranging from 20 to 60% (median 50%). The median dose was 12.5 Gy (9-15 Gy) and 20 Gy (12-28 Gy) for non-secretory and secretory adenomas, respectively. The median maximum dose to the optic chiasm was 3.7 Gy (0.1-8 Gy). and to the optic nerve was 3.4 Gy (0.2-7.6 Gy).

### Radiological evaluation

Tumor volume was assessed from the follow-up MRIs in 42 cases. RC was achieved in 41 (98%) cases (Table 1). Only one patient developed local tumor enlargement of more than 20% and, later, distant encephalic progression. This patient had an ACTH tumor that had failed after transsphenoidal, transcranial surgery, medication and EBRT. After RS, he was treated with adrenalectomy and developed Nelson's syndrome. He also underwent transcranial surgery that revealed pituitary carcinoma. This patient died 3 years after RS.

**Table 1:** Distribution of results of radiological evaluation

Radiological Evaluation	n	%
Stable	37	88
Progression	1	2
Response	4	10

Total	42	100

### Hormonal evaluation

Twenty-eight patients had functioning pituitary adenoma. HC was achieved in 22 (78%) cases. HN and HR were observed in 14 (50%) and 8 (28%) cases, respectively. Hormonal progression occurred in 1 case (Table 2).

**Table 2:** Distribution of the number and percentage of pituitary adenomas according to the diagnosis and hormonal evaluation

Diagnosis	Response	Normalization	Progression	Stable	Total (%)
ACTH	1	6	1	1	9 (32)
GH	4	4	0	2	10 (36)
PRL	3	4	0	2	9 (32)

Total (%)	8 (28)	14 (50)	1 (4)	5 (18)	28 (100)

The median pre and post-radiosurgical ACTH levels were, respectively, 102 and 47 pg/ml; the median pre and post-radiosurgical GH and IGF-1 levels were, respectively, 5.8 and 2.9 ng/ml and 688.5 and 361.5 ng/ml; the median pre- and post-radiosurgircal prolactin levels were, respectively, 55 and 25 ng/ml.

**Figure 1:**
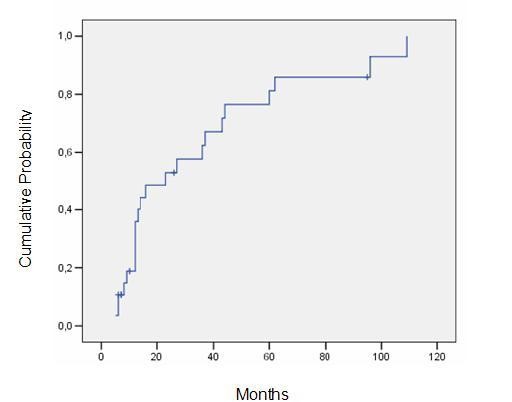
**Probability of hormonal control**.

**Figure 2:**
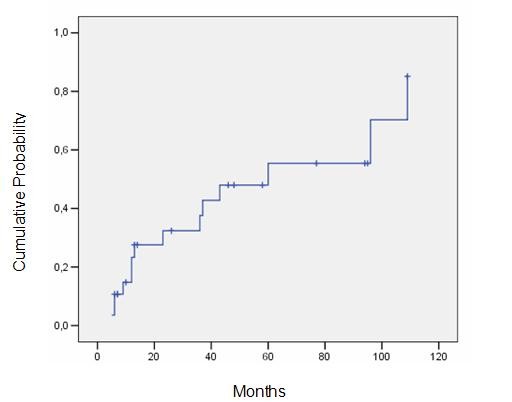
**Probability of hormonal normalization**.

The 3-year actuarial rate of HC and HN were 62,4% and 37,6%, respectively, and the 5-year actuarial rate were 81,2% and 55,4%, respectively (Figures 1 and 2). The median latency period for HC and HN was, respectively, 15 and 18 months (5-109 months).

On univariate analysis, there were no relationships between median dose or tumoral volume and HC or HN.

### Complications

There were no patients with visual deficit and 1 patient had hypopituitarism after RS. The patient who developed hypopituitarism after RS had the whole sella turcica defined as the target.

## Discussion

In the treatment of pituitary adenomas, radiotherapy is classically indicated in cases of incomplete resection or recurrent tumors, functioning tumors uncontrolled by medical therapy and patients inoperable or who refuse surgery. The objectives of radiotherapy are the control of tumor growth and/or the normalization of hormonal secretion, the maintenance of pituitary function and preservation of neurological function, especially visual acuity.

In a recent review, Prasad reported a control rate of tumor growth 67-100% with conventional radiotherapy [9]. Brada et al. reported tumor progression-free survival at 10 and 20 years of 94% and 89%, respectively [10].

The various retrospective series with RS published to date have shown the same results as conventional radiotherapy. Sheehan et al., in an extensive review of 1283 patients showed a mean tumor control rate of 96%. Considering only the series with mean or median follow-up of 4 years or more, the control ranged from 83 to 100%. Importantly, in all cases, control was defined as the persistence or reduction of tumor volume, as in our study [2].

The reduction in tumor volume, as observed in 4 cases in our series, is less than that is reported by others. Choi et al., after a mean follow up of 42.5 months in 42 patients with functioning adenomas also treated with the RS and a median marginal dose of 28.5 Gy, reported a growth control of 96.9% and a reduction in volume occurred in 40.6% of cases [11]. In this study, the reduction was also defined as a decrease greater than 20% tumor volume. Petrovich et al., also in a retrospective series of 78 patients treated only with the RS and a median prescribed dose of 15 Gy, reported a 96% tumor control, with volume reduction (> 50%) in 29% of cases after 36 months median follow-up [7]. Izawa et al., after mean follow up of 24 months in 79 patients, reported local tumor control in 93.6% of patients, with reduction in 24.1%. They prescribed a mean marginal dose of 22.5 Gy. The lower rate of tumor shrinkage in our series is probably related to a lower dose prescribed [12].

Probably even more important than the prescribed dose, the appropriate definition of the target volume is critical to the success of tumor control. For this, besides the careful evaluation of imaging studies, it is necessary to use greater amounts of information with respect to any prior surgeries performed. Meij et al. reported a higher incidence of reoperation in patients with dural invasion, indicating that it is an adverse prognostic factor for local control with surgery [13]. Likewise, it may be an adverse prognostic factor for RS.

The comparison of results between different series with RS becomes difficult due to wide variability of criteria for hormonal control and, sometimes, even the absence of defining a criterion. There is no consensus, for example, regarding the criteria for biochemical control of Cushing's disease. In patients with acromegaly, regardless of the definition of a gold-standard assessment for the evaluation of disease control, the control criteria in published studies vary depending on the practicality of the tests available. Only in patients with prolactinoma, the test is homogeneous [14].

In a review with a series of at least 10 patients and median follow up of 2 years, the rate of hormonal normalization ranged 17-83% in patients with Cushing's disease, 20-96% in patients with acromegaly and 0-84% in patients with prolactinoma [2]. In our study, we found hormone normalization in 67% of patients with Cushing's disease, 40% of acromegalic patients and 44% of patients with prolactinoma. If you also consider the patients who showed a reduction greater than 50% of hormone levels in relation to the value prior to radiosurgery (hormonal), we obtained a hormonal control of 77%, 80% and 77% respectively.

When we compare our results with recent retrospective series of patients treated with the RS and criteria for radiological control and hormonal defined and similar, we observe similar results.

Petrovich et al. reported a median time to normalization of hormonal 22, 18 and 24 months for patients with tumors that produce ACTH, GH and PRL, respectively. In our series, we observed a median time to hormonal normalization of 25, 18 and 24 months respectively [7]. Choi et al. reported a mean time to hormonal normalization of 21 months (2.8-59.1 months) and actuarial incidence of hormonal normalization at 1 and 3 years of 16.1% and 37.6%. In our study, the mean time to achieve hormone normalization was 33 months (5-109 months) and the actuarial incidence of hormonal normalization at 1 and 3 years was respectively 23.3% and 37.6% [11].

RS is possibly associated with a shorter latency period to achieve the hormonal control. Tsang et al. analyzed 145 patients with functioning adenomas after conventional radiotherapy and reported biochemical remission in 40% and, when considering those who still needed drug treatment after radiotherapy, 60% of patients over 10 years [15]. Landolt et al. compared 16 patients who underwent RS to 50 patients who underwent radiation therapy for acromegaly and persistent median time to normalization of GH and IGF-1 was 1.4 and 7.1 years respectively [8].

However, as well observed by Brada et al., the latency period to achieve the hormonal control is directly related to hormone level and therefore the tumor volume prior to treatment [16]. Considering that patients with large macroadenomas and considered unsuitable for RS for intimate relation to critical structures are usually selected to fractionated radiotherapy, it is expected that the time to normalize hormone would be higher in these cases. The most appropriate, then, would be to evaluate the time necessary for reduction to 50% of initial hormone level, what we defined as HR, and to consider it in the definition of HC. Choi et al. observed HR in 35 of the 42 patients (83.3%) and the mean duration between RS and HR was 6.8 months. In our report, 22 of the 28 patients (78%) with functioning pituitary adenoma achieved HC (all patients with HC had HR) and the median latency period for HC was 15 months [11].

It was not observed any relationship between the rate of hormonal control or normalization and tumoral volume and marginal dose in our series. However, Sheehan et al. has found an inverse correlation between marginal dose and time to endocrine remission and a direct correlation with control of adenoma growth. Besides that, smaller adenoma volume was correlated with improved endocrine remission [17].

Only one case of pituitary insufficiency induced by RS was observed in this series. As we did not have access to surveys of doses of various hormone sectors prior to and after RS in all patients, we chose to define pituitary insufficiency as the need for hormone replacement indicated by reference endocrinologist. This is a questionable criterion, because one does not detect patients who may be in the subclinical stage of hormone deficiency.

The incidence of hypopituitarism after RS reported in literature is quite variable. Older studies that included patients treated in the pre-computed tomography reported higher incidence. A retrospective study at the Karolinska Institute with a median follow up of 17 years showed an incidence of hypopituitarism of 72% [5]. More recent series have shown lower rates, with reported 0-36% incidence of hypopituitarism after RS [2].

Coupled with the relatively short follow-up, adopting a less objective criterion for the definition of hormonal sufficiency and a careful and conservative tactic in the contouring of structures and prescription of the dose required in most cases may explain the absence of hypopituitarism observed so far.

The absence of visual deficit induced by RS to date confirms the adequacy of indications of the procedures and plans made. More even than the concern about the pituitary function, we observe the maximum dose considered safe in the optic pathways and often used the blockage of collimators with plugs in order to optimize the planning and restrict the marginal dose prescribed. The median dose at the optic chiasm was 3.7 Gy (0,1-8 Gy).

Ideally, most studies suggest a maximum dose of 8 Gy to keep the risk of optic neuropathy close to zero and a minimum 2-5 mm between the tumor and optical apparatus [2,3,5]. However, in patients with functioning adenomas where the dose increase may be related to an increase in hormonal control, some authors accept the maximum dose of 10 Gy, since restricted to a small volume of the optical apparatus [18].

The multidisciplinary approach is directly related to therapeutic success in pituitary adenomas and, among treatment options for pituitary adenomas, RS has become increasingly evident. Our study showed that RS is an effective and safe method for obtaining tumoral and hormonal control and results overlapped with those of literature. Proper selection of patients, the careful definition of target volume and the respect to the dose tolerance of adjacent tissues are key factors to achieving these results.

## Conclusions

RS is an effective and safe therapeutic option in the management of selected patients with pituitary adenomas. The short latency of the radiation response, the highly acceptable radiological and hormonal control and absence of complications at this early follow-up are consistent with literature.

## Competing interests

The authors declare that they have no competing interests.

## Authors' contributions

DGC reviewed the medical records, performed the statistical analysis and wrote the manuscript. All authors attended patients, performed radiosurgery and read and approved the final manuscript.
